# The Acceptance and Use of the e-Health Instrument ‘The Personal Health Check’ in Four Dutch Municipalities: Lessons Learned

**DOI:** 10.1007/s10935-021-00651-2

**Published:** 2021-10-29

**Authors:** M. Rombouts, L. G. M. Raaijmakers, T. J. M. Kuunders, R. Van Steijn-Martens, T. de Vuijst, H. van Donkersgoed, L. A. M. van de Goor

**Affiliations:** 1 Municipal Health Service West-Brabant, Breda, The Netherlands; 2 Municipal Health Service Hart Voor Brabant, ‘S-Hertogenbosch, The Netherlands; 3 Netherlands Institute for Prevention and E-Health Development (NIPED), Amsterdam, The Netherlands; 4 Department Tranzo, Tilburg School of Social and Behavioral Sciences, Tilburg University, Tilburg, The Netherlands

**Keywords:** e-Health, Prevention, Primary care, Community approach, Healthy lifestyle

## Abstract

This pilot study assessed the acceptance and use of the e-Health instrument “the Personal Health Check” (PHC) among clients and professionals in primary care settings. By filling in the online PHC instrument, participants were provided insights into their health and lifestyle. When results revealed an increased health risk, participants were advised to undertake additional lab tests measuring blood pressure and haemaglobin levels. Based on the online questionnaire and optional lab tests, participants then received a report that included individually-tailored feedback from the e-Health application about personal health risks and suggestions for health interventions. The PHC was implemented in 2016 in four Dutch municipalities that determined which neighbourhood(s) the PHC targeted and how participants were invited. The Unified Theory of Acceptance and Use of Technology was used as a theoretical framework to address our research questions. Methods used to assess acceptance were: PHC instrument data, data from additional questionnaires completed by PHC participants, focus groups with PHC participants and professionals in primary care, and telephone interviews with non-responders to the invitation to participate in the online PHC. Of the 21,735 invited, 12% participated. Our results showed that participants and professionals in this pilot were predominantly positive about the PHC. Participants reported that they made an effort to apply the PHC lifestyle advice they received. Almost all had the knowledge and resources needed to use the PHC online instrument. Invitations from general practitioners almost doubled participation relative to invitations from the sponsoring municipalities. The overall low response rate, however, suggests that the PHC is unsuitable as a foundation on which to develop local public health policy.

## Introduction

An ageing population, lifestyle impairments, and an increasing number of patients with multiple morbidities are now considered to constitute major social and economic challenges (Cassell et al., 2018; Reeves et al., 2018). Chronic diseases, including heart disease, stroke, cancer, diabetes, and chronic lung disease, are collectively responsible for 71% of all deaths worldwide and thus impose the greatest burden on global health (World Health Organization, 2018). Research has shown that health care costs for chronic diseases in the Netherlands doubled between 2011 and 2016 (Nibud, 2016). According to the World Health Organization (2018), these chronic diseases would be mitigated by reducing four modifiable risk factors, namely smoking, excess weight, physical inactivity, and unhealthy diets. In 2017, 23% of the Dutch adult population 18 years and older smoked and 17% smoked daily (Rijksinstituut voor Volksgezondheid en Milieu, 2021). Half of the adult population was overweight (49%) and 14% were obese. Furthermore, in 2017 only 47% of Dutch adults met national physical activity guidelines. With regard to fruit and vegetable intake, 30% and 18% of adults, respectively, met established guidelines (Rijksinstituut voor Volksgezondheid en Milieu, 2021).

In recent years, primary care in the Netherlands has started to change its approach from one emphasizing ‘care and illness’ to one of ‘behaviour and health’ (Raad voor de Volksgezondheid & Zorg, 2010). Professionals in primary health care are encouraged to focus more on prevention and health promotion (Raad voor de Volksgezondheid & Zorg, 2010). Such a shift in primary care can make a difference in citizens’ awareness and their choices related to a healthy lifestyle (Zarrinkhameh, 2015). Primary care professionals increasingly acknowledge the advantages of this changing approach to health, but many lack appropriate tools to shift the focus to prevention (Geense et al., 2013). One promising approach to health promotion concerns the application of e-Health instruments developed to support behavioural change. E-Health applications can reduce healthcare costs, provide a higher quality of care, are easy to use, and increase access to healthcare for all individuals (Pagliari et al., 2005). E-health instruments can also contribute to the early detection of chronic diseases and are therefore important in the prevention of lifestyle-related diseases (Brunner-Ziegler et al., 2013; Damman et al., 2017). To account for local implementation challenges, tailoring interventions and providing personal feedback may increase their effectiveness (Storm et al., 2016).

Due to the growing number of similar e-Health applications, health professionals need a critical evaluation of their relative effectiveness. In addition, many e-Health interventions have been reported as failing during clinical implementation (Granja et al., 2018). Several sociodemographic factors, such as age, educational level, socio-economic status, and migration background, have been shown to affect the acceptance of general health checks (Brunner-Ziegler et al., 2013; Dryden et al., 2012). However, less is known about the adaptation and acceptance of e-Health instruments in primary care settings (Li et al., 2013). If these instruments are not well-received, it is unlikely that that they will be employed and sustained over time.

With this study, we sought to understand the acceptance and use among both the general adult population and among primary care professionals of an e-Health instrument called the Personal Health Check (PHC). In addition, we assessed the usability of PHC data for the development of local health policy. The PHC is a patented (Van Kalken & Kraaijenhagen, 2006) e-Health instrument, based on validated guidelines for personal health risk management. The PHC consists of different elements: an online questionnaire, additional lab tests (if applicable) and a personal health report. The PHC is developed by the Netherlands Institute for Prevention and E-health Development (NIPED). NIPED is a non-profit research institute that specializes in the development and application of e-Health in the area of individual prevention and early diagnosis in order to motivate people to make lifestyle changes (NIPED, 2021). The PHC seeks to increase awareness and motivation among participants and to prompt behaviours that are conducive to a healthier lifestyle (Colkesen et al., 2013).

In this pilot study, participants completed an online questionnaire that included questions about their socio-demographic characteristics, health-related behaviours, medical history, and psychosocial stress. When the results revealed an increased health risk, participants were advised to seek additional lab tests that measured blood pressure and blood levels (cholesterol, HbA1C, Hemoglobin). These tests were offered at so-called check-point locations in the participating municipalities. Participants could make an appointment within a few weeks after filling in the PHC. At the check-points, professionals of the municipal health service measured their blood pressure and took a blood sample from a finger-prick. The lab results were uploaded in the secure online PHC system where participants could access them through their personal account. Directly after completing the online questionnaire and/or receiving the results of the optional lab tests, participants received a personal health report in the secure online e-Health system with feedback about their personal health risks and tailored suggestions for locally available health interventions. For example, participants who did not meet the physical activity guidelines received suggestions for participating in physical activities in the neighbourhood, such as the local walking club. Tailored feedback is considered to be a successful way of promoting behavioural change (Krebs et al., 2010; Van den Brekel-Dijkstra et al., 2015).

In order to explain the acceptance and use of this novel intervention, we employed the Unified Theory of Acceptance and Use of Technology (UTAUT; Venkatesh et al., 2012). This theory is based upon the conceptual and empirical correspondence among eight theories and models of innovation acceptance and implementation (Venkatesh et al., 2003). The UTAUT model consists of two direct determinants of technology usage behaviour (intention and facilitating conditions), and three indirect determinants of usage (effort expectancy, performance expectancy, and social influence; Venkatesh et al., 2003). Subsequently, Venkatesh et al. (2012) incorporated price value (i.e. willingness to pay for technology use) as a sixth predictor of behavioural intention. The UTAUT model has demonstrated its usability in several studies on e-Health acceptance (Baumeister et al., 2014; Ebert et al., 2015; Li et al., 2013; Liu et al., 2014).

## Methods

### Design, Sample and Procedure

We used an observational design to assess the acceptance of the PHC by both participants within the general population and participating professionals in primary care. We implemented the PHC in 2016 in four municipalities in the province of North-Brabant in the Netherlands. The municipalities selected their own neighbourhood(s) for implementation and determined how participants would be invited (e.g., by primary care practices or by the municipality itself with support of the Municipal Health Service). Overall, 21,735 adults (aged ≥ 18) were invited to complete the online PHC questionnaire. The final response rate was 12.6% (2732 participants). In one municipality, the invitation was sent by a general practice instead of by the municipality. This doubled the response rate (22%) relative to that of the other three municipalities (12%, 10%, and 10%).

For this study, we used the anonymous online PHC questionnaire data provided by study participants to obtain insight in their health profiles (*N* = 2732). To assess acceptance of the PHC, we asked participants to complete three online follow-up questionnaires after 5 days (684 participants, 25%), 3 months (367 participants, 13%), and/or 6 months (441 participants, 16%). All participants had to complete the online PHC questionnaire to be invited for the subsequent three follow-up questionnaires. The estimated questionnaire completion time was 5 min for each of the three follow-up questionnaires. Because responses were anonymous, data from the PHC questionnaire and the three follow-up questionnaires could not be linked by participants across data collection waves. We therefore treated the follow-up questionnaires as independent samples. Participants gave written informed consent on the computer before they started filling in the PHC questionnaire according to data protection legislation. Ethical review was performed by Tilburg University.

To obtain more in-depth insights in the acceptance of the PHC among participants who completed the online PHC and to obtain some first insights into the acceptance among primary care professionals, we conducted a total of eight focus groups in the four participating municipalities: four with participants who completed the online PHC questionnaire (*n* = 25; between 5 and 7 in each group) and four focus groups with primary care professionals (*n* = 12; between 2 and 4 in each group). The focus groups were conducted after participants completed the online PHC questionnaire. Participants of the focus groups were recruited using different strategies in the four municipalities that included: advertisement in the local newspaper; advertisements on the websites of the municipalities; flyers and face-to-face recruitment at the primary care practices and at the checkpoints for additional lab tests; and word of mouth through social community teams, municipal health service employees, and neighbourhood councils. Primary care professionals were directly recruited by contacting participating primary care practices. The 2 h focus groups were semi-structured with open-ended questions, and followed a moderator’s guide based on the UTAUT model. The moderator’s guide for the focus groups with participants included the following topics: general experience with completing the online PHC, reasons for participation in the PHC, familiarity with the use of computers, websites, online questionnaires or apps; need for and received help with completion of the online PHC questionnaire; opinion about the invitation to participate in the online PHC; expectations about participating in the PHC; contribution of the PHC in improving health and lifestyle; participants’ health and lifestyle awareness; the role of the social environment in relation to participation in the PHC; willingness to pay a fee; recommendations for further implementation and reach of the PHC. The moderator’s guide for the focus groups with primary care professionals included the following items: general experience with participation in the PHC in primary care practices; perceived consequences (i.e., temporarily increased workload); expectations about the PHC; contribution of the PHC in improving health and lifestyle; insight into experiences of participants that completed the online PHC; attitude towards the PHC as e-Health instrument; willingness to pay a fee; recommendations for further implementation and reach of the PHC.

In addition to the focus groups, we conducted telephone and face-to-face interviews with 58 non-responders who did not participate in the online PHC questionnaire after receiving an invitation. The aim of these interviews was to obtain insight into the reasons for non-response. The non-responders interviews were conducted after the recruitment for completing the online PHC questionnaire was finished and parallel to the focus groups. Non-responders were recruited through an advertisement in the local newspaper, advertisements on the websites of the municipalities and face to face at the participating primary care practices. The 30-min interviews were semi-structured with open-ended questions, and followed an interview guide. Non-responders were asked whether they received the invitation to participate in the PHC and whether they visited the PHC website, their main reason for non-response, their impression of the invitation, conditions that would have led to participation and whether they discussed participation with their social environment.

All focus groups and interviews were conducted by the same facilitators. Participants consented to participate in the focus groups and non-responder interviews either in writing or verbally.

### Measures

The PHC questionnaire consisted of several measures. *Socio-demographic variables* (3 items) included age, sex, and educational level. *Health-related behaviours* included indicators of nutritional behaviour (4 items), physical activity (2 items), smoking behaviour (2 items), alcohol consumption (1 item). For each indicator, we used Dutch guidelines to determine whether participants met behavioural recommendations (Gezondheidsraad, 2015, 2017). *Body Mass Index (BMI;* 2 items) was calculated based on participants’ self-reported height and weight. *Cardiometabolic risk* was calculated using the algorithm developed by Alssema et al. (2012), a validated prediction tool to identify people who are currently free from disease but at high risk of future cardiovascular disease, Type 2 diabetes, or chronic kidney disease. Further, a *cardiovascular risk profile* was calculated based on the Systematic COronary Risk Evaluation project (SCORE; Conroy et al., 2003). This profile was calculated for participants who underwent additional lab tests and included the following risk factors: cholesterol level, age, sex, smoking and systolic blood pressure. *Psychosocial stress* was measured using four questions about stress at work and at home, financial stress, and major life events in the past year, which were based on the INTERHEART study (Rosengren et al., 2004). An example of an item was: ‘How often did you experience stress at home in the past year?’ (Response options: Never/sometimes/often/always/not applicable). *Vitality as a dimension of engagement* was measured with six items on a 7-point scale (1 = never to 7 = daily) based on the Utrecht Work Engagement Scale (Schaufeli, 2006) and adjustments were made for participants who were unemployed.

The online follow-up questionnaires included a relevant selection of seven determinants of the UTAUT model to assess acceptance of the online PHC (Venkatesh et al., 2012). Table 1 presents an overview of the measures included in each follow-up questionnaire. We measured all UTAUT items except for price value on a 4-point scale (1 = completely disagree to 4 = completely agree). *Price value* was measured by three items asking about participants’ willingness to pay for the PHC; if yes, how much; if no, why they were not willing to contribute.

**Table 1:** Description of the items of the online follow-up questionnaire at 5 days, 3 months, and 6 months after participation in the PHC

	5 days	3 months	6 months
*Performance expectancy*			
I expect that using the PHC will contribute to improvements in my health	x		
The PHC has contributed to improvements in my health			x
*Effort expectancy*			
The questionnaire was understandable	x		
The questionnaire was easy to follow	x		
The questionnaire was easy to fill in	x		
*Social influence *^***a***^			
People who are important to me think I should use the PHC		x	x
People who influence my behaviour think I should use the PHC		x	x
People whose opinions that I value prefer that I use the PHC		x	x
*Facilitating conditions *^*b*^			
I have the resources (like a computer, internet, Wi-Fi) necessary to use the PHC	x	x	
I have the knowledge necessary to use the PHC	x	x	
The use of the PHC is compatible with other websites, apps or computer programmes I use	x	x	
I can get help from others when I have difficulties using the PHC	x	x	
*Price value *^***c***^			
I am willing to pay for the PHC			x
*Behavioural intention or change *^***d***^			
Nutritional behaviour	x	x	x
Alcohol use	x	x	x
Smoking	x	x	x
Physical activity	x	x	x
Mental complaints	x	x	x
Physical complaints	x	x	x

^a^ Depending on the municipality, social influence was measured at the 3- or 6-month questionnaire

^b^ Facilitating conditions were measured at the 5-day questionnaire in three municipalities and for one municipality at the 3-month questionnaire

^c^ Price value was measured hypothetically compared to the concept in the UTAUT model because the application of the PHC was free for this study

^d^ Questions about behavioural intention (5-day questionnaire) and change (3-month and 6-month questionnaire) were only asked when a participant did not meet the norm for the particular lifestyle behaviour

### Data Analysis

We analysed PHC data and data from our follow-up questionnaires with SPSS version 21.0 and began by describing participant characteristics and findings related to our various outcome measures. Our logistic regression analyses employed the *Enter* method to determine correlates of BMI with sex or educational level as independent variables. We conducted similar logistic regression analyses with nutritional behaviour, physical activity, alcohol use, smoking, health risks and mental complaints as dependent variables. We performed linear regression analyses to determine whether lifestyle behaviour variables were associated with age. Associations were considered statistically significant if *p* < 0.05.

We audiotaped the focus groups with participants’ and professionals’ informed consent and transcribed a report for each group that was then sent by e-mail to participants for validation. Participants could sent their feedback by e-mail within a two-week period. We used the UTAUT model as the framework for qualitative analysis of the focus groups. Meaning units (i.e., words or sentences) were labeled with codes and grouped into categories and subcategories. The codes were checked by and discussed with a researcher and disagreements were resolved in a consensus meeting. Where data did not fit into the UTAUT model, we grouped them in additional categories, such as “reach of the PHC” and “other PHC-related issues”. Short reports were made of the telephone interviews with non-responders and the results were summarized for each interview question.

## Results

### Sociodemographic Characteristics

The mean age of participants who completed the online PHC questionnaire (*N* = 2732) was 54 years (range 19–97; *SD* = 13.7) and 56.3% of the participants were women. Low-educated participants were underrepresented (22.5%) relative to middle- (39.3%) and high-educated participants (38.1%).

### PHC Profile

Table 2 presents the PHC lifestyle profile of participants that completed the online PHC questionnaire and significant subgroup differences. Of the participants, 37.5% were overweight (excluding obesity) and 12.4% were obese and 74.4% met the physical activity standard. Furthermore, 14.5% smoked and 7.5% of the smokers, reported to smoke ten or more units of tobacco (cigarettes, shag, cigars, cigarillo and/or pipe) per day. Of the participants, 30% drank more than seven glasses of alcohol a week. Only 18.4% and 34.8% met the Dutch vegetable and fruit consumption standards, respectively. Of the participants with additional lab results (*n* = 668), 63.8% appeared to have an increased risk of cardiovascular diseases. Of those without additional lab results (*n* = 2001), 88.7% reported at least one risk factor for cardiometabolic diseases and 28.4% were being treated. In addition, 3.9% of all participants had diabetes, 57.1% had an increased risk of psychosocial stress, and 9.3% scored low on vitality, meaning they experienced less energy, less strength, and less (work) engagement.

**Table 2:** Lifestyle profile of participants as reported at baseline

Variable	%	*n*	Gender ^b^	Educational level^c^	Age (β)
*BMI*					
Underweight (< 18.5)	0.8	22	f > m*		− 0.028
Normal (18.5–25)	49.3	1314	f > m**	l < h,m**; h > l,m**	− 0.005**
Overweight (25–30)	37.5	1000	f < m**		0.004**
Obese (> 30)	12.4	331			0.004**
*Nutritional behaviour*					
Meets the Dutch vegetable standard (200gr a day)	18.4	489	f > m**		0.001
Meets the Dutch fruit standard (2 pieces a day)	34.8	925	f > m**		0.005**
Eats breakfast every day	81.8	2175	f > m**	l < m,h; h > l,m**	0.006**
Eats ≥ 1 fish a week	61.6	1636		m < l,h**; h > l,m**	0.007**
*Physical activity (PA)*					
Meets the Dutch PA guidelines (at least 5 days a week at least 30 min of moderate exercise) ^a^	74.4	1972	f > m**		0.002*
*Alcohol use*					
Drinks less than 7 glasses of alcohol each week	70.5	1545	f > m**	h < l,m**	− 0.005**
*Smoking*					
Smokes	14.5	385		l > m,h**; h < l,m**	− 0.003**
Smokes 10 or more units of tobacco each day	7.5	199		h < l,m**; l > h,l**	0.002
*Health risks*					
High risk on cardiometabolic disorders	88.7	1774	f > m*	l > m,h**; h < l,m**	0.004**
High risk on cardiovascular disease	63.8	426	f < m**		0.026**
Has been treated for a cardiometabolic disorder (Angina Pectoris, myocardial infarction, heart failure, stroke, hypertension, hypercholesterolemia, kidney disease)	28.4	757	f < m**	l > m,h**; m < l,h*; h < l,m**	0.011**
Has (had) diabetes mellitus	3.9	103	f < m**		0.002**
*Mental complaints*					
Has an increased risk on psychosocial stress (INTERHEART)	42.9	1146	f > m**	l < m,h	-0.010**
Vitality (as a dimension of engagement; UWES)	9.3	247		l > m,h**; h < l,m**	-0.001*

^*^p < 0.05. **p < 0.001

^a^ The calculation of the Dutch physical activity guidelines does not take into account the lower standard that applies to the 65 + age group

^**b**^ F = Female; M = Male

^c^ = primary school/basic vocational school; m = secondary vocational school/high school degree; h = higher educational degree/university degree

### Self-Reported (Intention to) Change Behaviour

Table 3 shows participants’ intention to change health-related behaviour directly after receiving personal feedback from the PHC application and their reported (intended) behavioural change at 5 days, 3 months, and then 6 months after participation. The immediate intention to change after completing the PHC was highest for losing weight (91.2%), followed by exercising more (73.5%), and quitting smoking (62.1%). For each lifestyle theme, the majority of the participants preferred to achieve their goals without professional help. Their intention to change 5 days after participation was the highest for nutritional behaviour and remained the highest both 3 and 6 months after participation. The majority of the participants who continued to take action to improve their alcohol intake (97.0%), nutritional behaviour (90.8%) and mental complaints (89.5%), reported making their changes without any professional help (data not shown). Physical activity changes were most often undertaken in a group (16.2%), and physical complaints were managed with professional help (39.5%; data not shown).

**Table 3:** Self-reported intention and behavioural change at baseline and at follow-up: 5 days, 3 months, and 6 months after participation in the PHC

Lifestyle themes	Intention online PHC-questionnaire			Intention 5-day questionnaire				Behaviour change 3-month questionnaire			Behaviour change 6-month questionnaire		
		Yes	No		Yes	Maybe	No		Yes	No		Yes	No
	*n*	%	%	*n*	%	%	%	*n*	%	%	*n*	%	%
Nutritional behaviour	2643	51.3	48.7	507	53.1	22.7	24.3	185	67.6	32.4	264	73.1	26.9
Alcohol use	382	37.2	62.8	284	28.9	51.4	19.7	80	40.0	60.0	164	38.4	61.6
Smoking	385	62.1	37.9	108	25.9	50.9	23.1	53	30.2	69.8	73	26.0	74.0
Physical activity	678	73.5	26.5	425	44.9	29.4	25.6	123	55.3	44.7	226	64.2	35.8
Losing weight	1580	91.2	8.8	–	–	–	–	163	58.3	41.7	226	61.5	38.5
Mental complaints	–	–	–	284	36.6	32.7	30.6	100	35.0	65.0	159	40.9	59.1
Physical complaints	–	–	–	291	49.5	27.1	23.4	86	37.2	62.8	160	46.9	53.1

*Note.* The four measurement times are treated as independent samples. Baseline PHC questionnaire *n* = 2732. Follow up questionnaires *n* = 1492

### Determinants of Acceptance

Table 4 shows the descriptive statistics of the UTAUT variables. Five days after baseline completion, 62% of the respondents expected that using the PHC would contribute to improving their health. After 6 months, almost half of the participants reported that the PHC had contributed to improving their health. Further, the majority of the participants thought that the questionnaire was understandable and easy to complete. The role of participants’ social environment in stimulating their use of the PHC was limited. The majority of the participants reported having the required facilitating conditions for using the PHC. While participants did not have to pay for the PHC, 56.7% reported they were not prepared to pay for the PHC, emphasizing that health insurance should take financial responsibility (data not shown). Other reasons given for participants’ unwillingness to pay for the PHC were that they did not see much added value in it or thought that they could monitor their health themselves. Of the participants, 5.7% said they would be willing to pay for the use of the PHC, in an amount that varied between €10 and €20 as a one-time cost.

**Table 4:** Descriptive statistics of acceptance of the PHC using the UTAUT model

UTAUT variables	*n*	(Completely) Disagree (%)	(Completely) Agree (%)
*Performance epectancy*			
I expect that using the PHC will contribute to improvements in my health	399	37.6	62.4
The PHC has contributed to improvements in my health	433	52.6	47.4
*Effort expectancy*			
The questionnaire was understandable	650	6.2	93.8
The questionnaire was easy to follow	650	6.7	93.3
The questionnaire was easy to fill in	650	10.6	89.3
*Social influence*			
People who are important to me think I should use the PHC	404	57.4	42.6
People who influence my behaviour think I should use the PHC	404	66.6	33.4
People whose opinions that I value prefer that I use the PHC	404	60.7	39.3
*Facilitating conditions*			
I have the resources (like a computer, internet, Wi-Fi) necessary to use the PHC	492	4.7	95,3
I have the knowledge necessary to use the PHC	496	3.0	97.0
The use of the PHC is compatible with other websites, apps or computer programmes I use	370	28.9	71.1
I can get help from others when I have difficulties using the PHC	282	25.9	74.1

*Note.* Response options for each item were (1) completely disagree, (2) disagree, (3) agree, (4) completely agree. Answer options were dichotomised as (completely) disagree = responses 1 + 2, and (completely) agree = responses 3 + 4

Among the participants who completed the questionnaire after 5 days (*n* = 395), 51.4% reported that they would participate again in the PHC, and 20.8% would consider doing so. In the 3- and 6-month questionnaires, 57.8% and 45.2% of the participants, respectively, were willing to participate again. Of the participants that filled in the questionnaire after 3 months, 21.7% had contacted a local professional (e.g., dietician, physiotherapist), as recommended in their personal health report.

### Focus Groups Results

#### Performance Expectancy

Three quarters of participants of the focus groups reported that their PHC results matched their expectations and were mainly a confirmation of the healthy aspects of their lifestyle and the health risks with which they were already familiar. About a quarter of the participants said they critically evaluated the advice provided in their personal health report. For example, one focus group participant said: *“You have to maintain a critical attitude to determine whether the results and advice apply to your personal situation and keep interpreting yourself.”*

Three quarters of professionals from the four focus groups reported that the PHC had brought relatively limited new insights. Contrary to their expectations, only a few new patients were diagnosed with chronic conditions, and the PHC did not lead to an increased workload through an increase in patients visiting general practitioners with questions or concerns. About a quarter of the professionals and participants wrongly expected that the PHC would provide their general practitioners (GP) with automatic feedback about their patients’ results: *“I expected to automatically obtain sight of the results of patients who have increased health risks, but who rarely or never come to my practice.”* According to these participants and professionals, this would be of added value and could increase the effect of the PHC.

#### Effort Expectancy

Almost all focus group participants experienced the PHC as user friendly and understandable. However, they reported that some questions were difficult to understand and that answer options did not always accommodate their personal situation.

The majority of the professionals in the focus groups evaluated the PHC as a valid instrument that could enhance consciousness among participants and could contribute to public health, especially when it reached people who are not regularly seen in primary care. However, about a quarter of the professionals found the questions to be too extensive, a possible barrier to participation: *“The PHC is not easily accessible and I wonder whether you miss certain groups with this instrument.”* These professionals suggested a reconsideration of the questions asked and advice given since they believed those could be misunderstood.

#### Social Influence

Almost all participants of the focus groups were not influenced by their social environment to use the PHC. Only a few participants discussed the PHC pilot with other people: “*Different people in my neighbourhood shared their doubts about the invitations sent by the municipality and had privacy-related objections.”*

#### Facilitating Conditions

All focus group participants reported possessing the knowledge and resources to use the PHC: *“The PHC was easily accessible and we are familiar with the use of digital applications.”* Three-quarters of participants thought the PHC was not suitable for elderly people who, they claimed, are often unfamiliar with digital systems. About a quarter of the professionals reported that participants stopped using the PHC because of language difficulties or barriers related to using the internet.

#### Price Value

In general, almost all participants in the focus groups were willing to pay a fee for the PHC, although about a quarter thought the response would be better if the PHC were subsidised by the government, paid by insurance. Moreover, several wondered whether a fee was necessary, since preventive activities would eventually lead to lower health care costs.

All professionals thought that charging a fee to participate in the PHC would be a barrier, especially for lower educated people, and would likely yield a select group of participants, thereby increasing health inequities.

#### Reach of the PHC

Overall, participants said that an invitation sent by their GP would create more trust and lead to a better response than would an invitation from their municipality. *“The GP invitation creates more urgency and trust. The invitation from the municipality is more non-binding.”* However, a few participants thought it was helpful that the municipalities were involved in the pilot.

The health care professionals concurred. They believed that people had more confidence in their GP in health matters and regarded an invitation from the GP as more personal than an invitation from their municipality.

#### Results from Interviews of Non-Responders

The interviews with non-responders revealed that the main reasons for non-response were that people were already under the care of their GP or specialist, and that they had already received an annual check-up through their GP or employer. Privacy-related issues were a further reason for non-response, since people expressed a desire to only share confidential health-related information with their health care providers.

## Discussion

Our study assessed the acceptance and use of the PHC among participants and primary care professionals, as well as the utility of PHC data as a basis for developing local health policy.

Regarding the acceptance of the PHC, our results (online follow-up questionnaires and focus groups) show that participants and primary care professionals were predominantly positive. In addition, participants reported applying the lifestyle advice provided by the PHC, or at least making an effort to do so. More insight into their personal health data should also encourage people to take increasing responsibility for their health, since it should keep them empowered and actively engaged both in their health and in the prevention or progression of further disease (Fylan et al., 2018; Lucivero, 2017; Niezen & Verhoef, 2018). Often, e-Health services are not adopted because of a lack of acceptance by their users (Choi et al., 2019). A recent review by Schreiweis et al. (2019) reported limited exposure to and knowledge of e-Health (i.e., poor digital health literacy) as the most frequently reported barrier, followed by the lack of devices needed for access, together with problems related to costs. In this pilot, the PHC was provided for free, and both participants and professionals reported that paying a fee to use it would be a barrier to acceptance. Coverage by health insurance or through employers might overcome this barrier.

Our results concerning participants’ intention to change their behaviours and action undertaken, as characteristics of acceptance, indicate that the PHC appeared to create awareness of participants’ health and health related behaviour and motivated a large group of participants to make an effort to improve their health. In general, e-Health seems to stimulate patients’ self-management and empowerment by providing them with more knowledge about their disease or lifestyle and increases their receptivity to, involvement in, and maintenance of a healthier lifestyle (Talboom-Kamp et al., 2018; Van der Kleij et al., 2018). Actual behavioural change as reported in our study at either 3 or 6 months follow-up varied for the different lifestyle behaviours we specified. It has been widely demonstrated that intentions to change behaviours do not guarantee actual behavioural change (Webb & Sheeran, 2006). Evidence suggests that intentions are translated into action about half of the time (Webb & Sheeran, 2006).

Purposeful guidance toward the PHC appeared to be an important and necessary first step to a healthier lifestyle for participants. Our printed invitation signed by the GP doubled the response rate relative to the invitation from the municipality. This suggests that a personal and professional recommendation from a GP, as a highly rated professional (Schäfer, 2016), is an important success factor that can be used to reach the target population. This can be attributed to the specific patient-GP relationship and patients’ expectations of their GP’s role (TNS NIPO, 2011). Furthermore, the quality of the contact between doctor and patient can influence how a patient responds to illness and treatment (Chandra et al., 2018; Kelley et al., 2014; Tarrant et al., 2003).

We also found that participants in this pilot had an above-average healthy lifestyle profile relative to the Dutch population in general. However, two thirds did not meet national standards for alcohol use, nor for fruit and vegetable intake. As reach can be considered a key aspect of ‘acceptance,’ an important question is whether the PHC, as implemented in this pilot, reached individuals at higher risk-levels, where the greatest health gains can be achieved. People with a less healthy lifestyle are more often found among groups with lower education, lower income levels, and migratory backgrounds (Pharos, 2019). Insufficient health and e-Health literacy both play an important role in this respect, as many from lower socioeconomic status groups experience difficulties in understanding health-related information. The first European health literacy survey among eight European countries showed that 28.7% of the Dutch participants had difficulty understanding health related information (Sørensen et al., 2015). Subgroups within the population, as defined by financial deprivation, lower social status, lower education, or older age, had higher proportions of people with limited health literacy (Sørensen et al., 2015). Regarding e-Health literacy, almost all participants in our study reported having the knowledge and resources needed to use the PHC, which is in line with previous research (Ozok et al., 2014), and indicates a sufficient level of e-Health literacy.

Our study’s low and selective response emphasizes that increasing the response rate should be specifically addressed in future when implementing the PHC, particularly within low socio-economic groups. In a study implementing a precursor of the PHC in the Netherlands, a much higher response rate (70%) in underserved populations was achieved by a culturally adapted postal approach initiated by respondents’ GPs with a follow-up call to non-responders (Groenenberg et al., 2015). Research showed that, in general for optimal use of e-Health, different stakeholders such as patients, entrepreneurs, health care professionals and health care insurers should collaborate (Swinkels et al., 2018; Van der Kleij et al., 2018; Versluis et al., 2020). They should preferably be identified and involved at an early stage in the development and implementation of e-Health interventions (Nilsen et al., 2020). Our study emphasizes the value of professionals in the primary care practice as stakeholders, since the GPs’ invitation almost doubled participation in one of our municipalities. This finding underscores the importance of “blended care,” in which face-to-face care is combined with e-Health applications (Van der Kleij et al., 2018). This approach could be used to expand the role of the primary care practices, by referring to the PHC in routine care contacts and discussing the lifestyle advice generated, which may facilitate reaching underserved populations.

Some focus group participants reported that they felt the results and advice received in the health report were a poor fit for their personal situation. Although tailored feedback was provided after participants completed the online PHC, some revisions might be needed to make sure the feedback fits better to the target group since tailored feedback is important in promoting healthy behaviour (Kaptein et al., 2015; Krebs et al., 2010). Finally, the involvement of end users such as patients and their caretakers, consumers, and healthcare professionals would increase the likelihood that the development and implementation of digital health solutions would be driven by people’s practical health needs (European Public Health Alliance, 2017; Haluza & Jungwirth, 2015).

### Limitations

We acknowledge a number of limitations to our study. Our findings are based on self-reported data, which may have led to socially desirable responses. Furthermore, people tend to underestimate unhealthy behaviours (Prince et al., 2020). In addition, the determinants of acceptance based on the UTAUT model were measured in separate questionnaires completed by different participants. Participation was anonymous, which means that the data from the three follow-up time periods could not be linked to any given individual. We therefore only reported descriptive results of the three follow-up questionnaires. We did not include results concerning changes over time, since we cannot base such conclusions on independent samples that differed significantly on some demographic characteristics of participants. This study did not provide deeper insight into how participants undertook action and to what effect. Nor do we know whether any reported intended behaviour change may have occurred without participation in the PHC, since this study did not include a control group (Reinwand et al., 2015). Further, some measures of several determinants of the UTAUT model needed to be adjusted for this pilot study because of the limited length of the questionnaires. We did not include two determinants of the UTAUT model in this study: *habit,* as the PHC was implemented as a pilot and the period too short to measure this variable, and *hedonic motivation,* because it appeared less relevant to us and the length of the questionnaire was limited. People with a lower educational level are less likely to make use of e-Health applications (Latulippe et al., 2017). The fact that this group was underrepresented in our sample is a limitation.

Despite the positive influence of the GP invitation in one of the municipalities, the overall response rate to the invitation to complete the online PHC questionnaire was very low compared with the response on regular health monitors conducted by the municipal health services among the general Dutch population every four years (12.6% of invited participants in the PHC vs. average 30.8% of invited adults in the health monitors). When we compare the background characteristics of participants with national and municipal populations, less-educated people, people between 19 and 35 years old and people with an unhealthier lifestyle were underrepresented in our sample (Centraal Bureau voor de Statistiek, 2018).

## Conclusion

The participants and main stakeholders involved in this study were predominantly positive about the PHC. Almost all participants had the knowledge and resources needed to use the PHC online instrument. Invitations by GPs relative to municipalities almost doubled participation. However, the study’s overall low response rate makes data from the PHC unsuitable as a basis on which to develop local public health policy.
